# Supplementary material for: SARS-CoV-2 papain-like protease activates nociceptors to drive sneeze and pain
Source: bioRxiv. 2024 Jan 11:2024.01.10.575114. Preprint. [Version 1] doi: 10.1101/2024.01.10.575114 (PMC10802627; doi:10.1101/2024.01.10.575114)
Supplement: 1 [file NIHPP2024.01.10.575114v1-supplement-1.pdf]

bioRxiv preprint doi: https://doi.org/10.1101/2024.01.10.575114; this version posted January 11, 2024. The copyright holder for this preprint (which was not certified by peer review) is the author/funder, who has granted bioRxiv a license to display the preprint in perpetuity. It is made available under aCC-BY 4.0 International license.

SARS-CoV-2 papain-like protease activates nociceptors to drive sneeze and pain

Sonali S. Mali[1,2], Ricardo Silva[1], Zhongyan Gong[1,2], Michael Cronce[3], Uyen Vo[1,4], Cliff Vuong[1], Yalda Moayedi[5], Jeffery S. Cox[1], Diana M. Bautista[1,2,4*]

[1]Department of Molecular and Cell Biology, University of California, Berkeley, Berkeley, CA
[2]Helen Wills Neuroscience Institute, University of California, Berkeley, Berkeley, CA
[3]Department of Bioengineering, University of California, Berkeley, Berkeley, CA
[4]Howard Hughes Medical Institute
[5] Pain Research Center, Department of Molecular Pathobiology, New York University College of Dentistry, New York, NY

*Correspondence should be addressed to: dbautista@berkeley.edu

bioRxiv preprint doi: https://doi.org/10.1101/2024.01.10.575114; this version posted January 11, 2024. The copyright holder for this preprint (which was not certified by peer review) is the author/funder, who has granted bioRxiv a license to display the preprint in perpetuity. It is made available under aCC-BY 4.0 International license.

Abstract

SARS-CoV-2, the virus responsible for COVID-19, triggers symptoms such as sneezing, aches and pain.[1] These symptoms are mediated by a subset of sensory neurons, known as nociceptors, that detect noxious stimuli, densely innervate the airway epithelium, and interact with airway resident epithelial and immune cells.[2–6] However, the mechanisms by which viral infection activates these neurons to trigger pain and airway reflexes are unknown. Here, we show that the coronavirus papain-like protease (PLpro) directly activates airway-innervating trigeminal and vagal nociceptors in mice and human iPSC-derived nociceptors. PLpro elicits sneezing and acute pain in mice and triggers the release of neuropeptide calcitonin gene-related peptide (CGRP) from airway afferents. We find that PLpro-induced sneeze and pain requires the host TRPA1 ion channel that has been previously demonstrated to mediate pain, cough, and airway inflammation.[7–9] Our findings are the first demonstration of a viral product that directly activates sensory neurons to trigger pain and airway reflexes and highlight a new role for PLpro and nociceptors in COVID-19.

bioRxiv preprint doi: https://doi.org/10.1101/2024.01.10.575114; this version posted January 11, 2024. The copyright holder for this preprint (which was not certified by peer review) is the author/funder, who has granted bioRxiv a license to display the preprint in perpetuity. It is made available under aCC-BY 4.0 International license.

Main

Common symptoms of viral infections including SARS-CoV-2, the virus responsible for COVID-19, include sneezing, headache, and other aches and pain.[1,10] Sneezing is a common host response to SARS-CoV-2 infection that promotes transmission by generating and spreading airborne droplets that contain the virus. Somatosensory and vagal sensory neurons drive protective reflexes, including sneezing, and initiate pain sensations during injury or infection.[5,11] Yet, little is known about how these neurons are activated by SARS-CoV-2 infection to drive symptoms. As epithelial cells are infected, they release cytokines and other inflammatory mediators that activate surrounding epithelial and immune cells, as well as the sensory neurons that densely innervate the epithelia. Studies to date have focused on endogenous inflammatory mediators released during viral infection that activate sensory neurons.[12–15] However, infection also generates viral proteins that may be released and serve as direct host modulatory factors. Viral products are known to activate airway epithelial and immune cells to drive inflammation, but whether viral molecules act directly on sensory neurons to drive pain and airway reflexes is unknown.[16,17] Among viral molecules, viral proteases have been of much interest as therapeutic targets for COVID-19 due to their critical role in viral replication. While proteases from plants, insects, bacteria, and immune cells have been demonstrated to activate sensory neurons to trigger itch, pain and inflammation;[18–27] viral proteases in this context have been unexplored. Here, we show a new role for the SARS-CoV-2 papain-like protease (PLpro) in the activation of airway-innervating sensory neurons.

PLpro plays an essential role in viral replication by processing viral polyproteins and suppressing the host immune response of infected cells.[28,29] Can active PLpro be released from infected cells and thus be poised to activate surrounding uninfected cells? To test this, we infected human airway epithelial cells with SARS-CoV-2 and measured PLpro activity in the supernatants of infected cells. We detected PLpro activity 48 hours but not 24 hours following infection despite observing comparable level of viral transcripts 24 and 48 hours post infection (Fig. 1a, b). This difference may reflect an increase in protein translation and accumulation over time and/or the initiation of the mechanisms that release PLpro, such as lytic cell death. We find that PLpro is released at nanomolar concentrations from infected airway epithelial cells (Fig. 1c), and is therefore positioned to act on neighboring cells, such as the free nerve endings of sensory neurons in the airways.

We next used functional imaging to test whether PLpro could activate the sensory neurons that densely innervate the epithelial cells of the nasal mucosa and upper airways. We recorded calcium responses in the trigeminal ganglion (TG) from mice expressing the calcium indicator (GCaMP6s) in sensory neurons to intranasal perfusion of vehicle, PLpro (10 µM), the TRPA1 channel agonist, allyl isothiocyanate (AITC; 1 mM), and the TRPV1 channel agonist, capsaicin (100 µM; Fig. 2a-c).

bioRxiv preprint doi: https://doi.org/10.1101/2024.01.10.575114; this version posted January 11, 2024. The copyright holder for this preprint (which was not certified by peer review) is the author/funder, who has granted bioRxiv a license to display the preprint in perpetuity. It is made available under aCC-BY 4.0 International license.

Strikingly, PLpro elicited rapid and robust calcium transients in 37.8% of recorded neurons (Fig. 2c-e). Trigeminal sensory neurons are heterogenous and transduce a range of modalities such as pain, itch, temperature, and touch. Thus, as expected, we observed both distinct and overlapping subsets of these neurons that respond to intranasal perfusion of various compounds (Fig. 2b-d). Intranasal perfusion of vehicle alone elicited a transient increase in calcium in a subset of recorded neurons (36.4%; Fig. 2e). The subsequent perfusion of PLpro induced calcium transients in 28.6%of these vehicle-sensitive neurons and recruited an additional 27.4% of neurons (Fig. 2d). Subsequent addition of AITC and capsaicin activated 34.4% and 47.2% of neurons, respectively (Fig. 2e). Subsets of sensory neurons that express the ion channels TRPV1 and TRPA1, known as nociceptors, trigger pain and defensive reflexes in response to a variety of noxious stimuli, including mechanical stimuli, chemical irritants, and inflammatory mediators. We found that PLpro (but not vehicle) activated 15.9% of TRPV1-expressing and 18.4% of TRPA1-expressing neurons (defined by their capsaicin and AITC responsiveness, respectively; Fig. 2f, g). In a separate control experiment, we perfused vehicle twice; perfusion of this second vehicle activated only 2.7% of TRPV1-expressing and 9.1% of TRPA1-expressing neurons (Fig. 2f, g). Taken together, these data demonstrate that PLpro activates a subset of airway-innervating nociceptors in vivo.

Trigeminal nociceptors mediate sneezing[2], nasal secretion[30], and orofacial pain[31], which are common symptoms of viral infection. Which, if any, of these behaviors does PLpro trigger? We used high speed video and audio recordings to quantify behavioral responses to intranasal treatment of vehicle or PLpro (10 μM). PLpro elicited rapid sneezing (latency: 13.6 ± 4.1s) over two minutes in mice (Fig. 3a-c, Supplemental Fig. 1i). In contrast, vehicle-treated mice were slower to sneeze (latency: 30.5 ± 12.1s) and sneezed less (Fig. 3b, c, Supplemental Fig. 1i). The sneeze reflex involves the convulsive expulsion of air to clear the airways of harmful irritants and pathogens. To visualize nasal expulsion, we also applied PLpro or vehicle in combination with blue dye. The number of sneezes, quantified from the audio recordings, correlated with dye on the bottom of the chamber (Supplemental Fig. 1a-c). We occasionally observed the expulsion of dye onto the side of the chamber and the floor during these audible events (Fig. 3a, Supplemental Fig. 1b). These observations show that PLpro triggers rapid sneezing and nasal secretion that could expel SARS-CoV-2 from the nasal cavity. Previous studies have shown that irritants and inflammatory mediators such as capsaicin, histamine, serotonin, and allergens can drive sneezing in mice.[2,32,33] This data demonstrates for the first time that a viral molecule, PLpro, is alone sufficient to trigger sneezing, a key symptom of viral infection and mechanism for viral spread.

bioRxiv preprint doi: https://doi.org/10.1101/2024.01.10.575114; this version posted January 11, 2024. The copyright holder for this preprint (which was not certified by peer review) is the author/funder, who has granted bioRxiv a license to display the preprint in perpetuity. It is made available under aCC-BY 4.0 International license.

We also observed a nocifensive behavior, nose rubbing, characterized by a fast elliptical stroke of the front paws around the nose (Fig. 3d, Supplemental Fig. 1d, e). These nose rubs are distinct from face wiping (a stroke of the front paw around the face) such as those triggered by injection of algogens to the cheek.[34] Unlike grooming behaviors, the initial nose rubs were not followed by wiping or body licking (Supplemental Fig. 1e,f). Intranasal PLpro evoked the first nose rub significantly faster (23.1 ± 3.3 s) than in vehicle-treated mice (42.3 ± 6 s); though, PLpro-treated mice did not rub any more than vehicle-treated mice over two minutes (Fig. 3e, Supplemental Fig. 1g). Interestingly, we observed a dose-dependence of these behaviors such that a lower dose of PLpro (100 nM) triggered fast sneezing (latency: 16.1 ± 5.1 s) but not a shorter latency to nose rubbing (latency: 39.9 ± 12.0 s) whereas the higher dose of PLpro (10 μM) rapidly elicited both a sneeze and nose rub (Supplemental Fig. 1i-l). Moreover, the co-occurrence of acute rubbing and sneezing in the first 30 seconds was dose-dependent (vehicle: 9%, 0.1 μM PLpro: 41.7%, 10 μM PLpro: 55%; Fig. 3f; Supplemental Fig. 1j-l).

In addition to triggering airway reflexes, activation of distinct subsets of trigeminal nociceptors also promotes itch or pain behaviors. Thus, we examined the consequences of PLpro in a cheek injection model, where painful stimuli promote wiping with the forelimbs and itch stimuli evoke scratching with the hindlimb. Mice injected with PLpro displayed a significantly shorter latency to wipe (vehicle: 152.6s, PLpro (10 μM): 56s, PLpro (50 μM): 29.6s; Supplemental Fig. 2a) and wiped more often than vehicle injection (vehicle: 0, PLpro (10 μM): 0.56, PLpro (50 μM): 1.3 wipes; Supplemental Fig. 2b). In contrast, PLpro did not elicit itch-evoked scratching behaviors (Supplemental Fig. 2c, d). Overall, this data indicates that PLpro drives acute site-directed behaviors associated with pain and irritation and, interestingly, triggers a faster and more robust response in the nasal cavity than in the skin.

To elucidate the mechanisms by which PLpro activates nociceptors, we tested if the protease could directly activate sensory neurons in vitro. The airways are innervated by somatosensory neurons from the TG, dorsal root ganglia (DRG), and vagal sensory neurons from the nodose and jugular ganglia (NJG). We found that PLpro induced calcium transients in neurons cultured from all three ganglia from mice (Fig. 4a, b, f). We observed rapid and robust calcium transients and a dose-dependent increase in percentage of neurons responding to PLpro in TG cultures (1 nM: 3.8%, 10 nM: 7.2%, 100 nM: 9.3%; Fig. 4a, f). We observed similar proportions of responses in DRG cultures (Veh: 2.9%, 1 nM: 4.2%, 10 nM: 6.8%, 100 nM: 11.9%; Fig. 4b). Interestingly, we observed a greater fraction of neurons that responded to PLpro in cultures of NJG neurons across all PLpro concentrations (Veh: 5.5%, 1 nM: 21.7%, 10 nM: 23.6%, 100 nM: 33.9%; Fig. 4b). PLpro-responsive neurons were of smaller diameter than vehicle-responsive neurons and the population overall (Supplemental Fig. 3a-c).

bioRxiv preprint doi: https://doi.org/10.1101/2024.01.10.575114; this version posted January 11, 2024. The copyright holder for this preprint (which was not certified by peer review) is the author/funder, who has granted bioRxiv a license to display the preprint in perpetuity. It is made available under aCC-BY 4.0 International license.

Consistent with the activation of a subset of nociceptors in vivo, nearly all PLpro-responsive neurons also responded to capsaicin (DRG: 85.7%; NJG: 100%) and a subset also responded to AITC (DRG: 33.3%, NJG: 50%, Fig. 4c). As observed in mouse neurons, PLpro also evoked calcium transients in a subset of human iPSC-derived nociceptors (Fig. 4d). Of the responders, 16.7% responded to vehicle, 50.0% to PLpro, and 33.3% to capsaicin. PLpro activated a distinct subset than vehicle and elicited significantly more calcium influx than vehicle (Fig. 4e). Taken together, we demonstrate that PLpro directly activates a subset of human and mouse nociceptors that express the TRPA1 and TRPV1 ion channels.

We further probed the mechanism by which PLpro activates nociceptors in mice. We observed little response to PLpro in the absence of extracellular calcium (control: 5.5% vs. EGTA: 1.2%; Fig. 4i) suggesting that the activation of neurons by PLpro requires a calcium-permeable ion channel. Airway irritants and inflammatory mediators, including proteases, activate nociceptors via the calcium-permeable ion channels, TRPV1 or TRPA1.[7,35–38] To test the requirement of these channels, we examined PLpro responses in TRPV1 or TRPA1 deficient mice and found that the percent of PLpro responders was significantly reduced (WT: 5.5%, Trpv1 KO: 2.4%, Trpa1 KO: 1.9%; Fig. 4e). The acute blockade of TRPA1 with the antagonist HC-030031 also reduced PLpro responders (HC: 1.7%) comparable to responses observed in Trpa1 KO neurons (Fig. 4e). These data demonstrate that PLpro activates sensory neurons via TRPA1 and TRPV1 ion channels. However, the mechanism by which PLpro activates neurons via these channels remains unclear. Neither human nor mouse TRPV1 or TRPA1 contain the cleavage sequence for PLpro, and thus, it is likely that PLpro, like other proteases, may be activating a receptor that is coupled to TRPV1 and TRPA1.[39]

PLpro is expressed by all coronaviruses including the pathogenic betacoronaviruses, SARS-CoV (SARS) and MERS-CoV (MERS) that share 89% and 51% sequence similarity to SARS-CoV-2 PLpro, respectively.[28] While PLpro from these three coronaviruses share nearly identical substrate cleavage sequences, previous studies have shown differences in the structure and catalytic efficiency of MERS PLpro compared to SARS PLpro and SARS-CoV-2 PLpro.[40,41] Thus, we assessed whether the SARS and MERS PLpro activate mouse neurons. SARS PLpro also activates subsets of neurons in a dose-dependent manner (Fig. 4g), whereas few neurons were activated by MERS (Fig. 4h). It will be interesting to assess whether PLpro from other viruses such as the common cold can also activate neurons or whether this is unique to SARS and SARS-CoV-2 PLpro.

bioRxiv preprint doi: https://doi.org/10.1101/2024.01.10.575114; this version posted January 11, 2024. The copyright holder for this preprint (which was not certified by peer review) is the author/funder, who has granted bioRxiv a license to display the preprint in perpetuity. It is made available under aCC-BY 4.0 International license.

Recent studies have shown that SARS-CoV-2 infects sensory ganglia in human, hamster, and mouse and can directly infect human iPSC-derived nociceptors. While these neurons are susceptible to infection, unlike airway epithelial cells, the virus does not replicate.[42–44] Despite this lack of replication, infection may modulate neuronal physiology. Thus, we set out to assess the consequences of SARS-CoV-2 infection on cultured mouse trigeminal ganglia. We detected SARS-CoV-2 viral transcripts 48 hours post infection in cultured mouse TG neurons including the transcript *ORF1ab* that encodes PLpro (Fig. 5a). These data suggest that in addition to airway epithelial cells, neurons may be an additional source for PLpro.

What are the consequences of SARS-CoV-2 infection on sensory neuron gene expression? SARS-CoV-2 infection significantly upregulated 789 genes and downregulated 1664 genes with the most differentially expressed genes being related to inflammation (Supplemental Fig. 4a, b). We then assessed how infection and this infection-induced inflammatory response alters the transcriptional signatures of distinct functional neuronal subtypes within the sensory ganglia. Nociceptor-associated gene sets were significantly altered, including those corresponding to both non-peptidergic and peptidergic nociceptors, while gene sets associated with mechanoreceptors, proprioceptors, and itch were not significantly changed (Fig. 5b). This suggests that infection drives transcriptional changes that selectively impact nociception. More so, several of the most upregulated nociceptive genes encode ion channels (*P2rx3, Scn9a, Trpa1, Trpv1, Cacna1b, Cacna1a, Chrnb2,* and *Grin1*) that can increase the excitability of nociceptors to promote pain (Fig. 5c). Interestingly, a previous study showed that human rhinovirus infection of human neuroblastoma cells also upregulated *TRPV1* and *TRPA1*.[45] Here, we demonstrate that the genes for *Trpv1* and *Trpa1* channels are both upregulated by SARS-CoV-2 infection and are required for acute PLpro-evoked neuronal activation (Fig. 4i, 5c). SARS-CoV-2 infection in vivo also drives gene expression changes in hamster sensory ganglia that are related to neuronal signaling and neuroinflammation,[46] which could reflect consequences of the systemic response to infection or direct infection of the ganglia. Our in vitro data support a model in which direct infection of the ganglia can drive gene expression changes to alter nociceptor function and promote inflammation.

A key feature of TRPA1- and TRPV1-expressing neurons is the release of the neuropeptides calcitonin gene-related peptide (CGRP) and Substance P that can drive neurogenic inflammation[47] and immunomodulation.[5,48] We first tested if PLpro alone was sufficient to elicit release of CGRP and Substance P from trigeminal ganglia cells in vitro. PLpro triggered acute release of CGRP (Fig. 5d,). However, surprisingly, unlike many allergen proteases, we did not observe the release of Substance P (Fig. 5e).[19,20] We next tested whether PLpro could stimulate the nerve endings of sensory neurons

bioRxiv preprint doi: https://doi.org/10.1101/2024.01.10.575114; this version posted January 11, 2024. The copyright holder for this preprint (which was not certified by peer review) is the author/funder, who has granted bioRxiv a license to display the preprint in perpetuity. It is made available under aCC-BY 4.0 International license.

to release CGRP. Using an ex vivo mouse trachea model, we found that PLpro, but not vehicle treatment, induced significant release of CGRP above baseline (Fig. 5c). These data demonstrate that activation of neurons by PLpro is sufficient to induce acute release of CGRP both in vitro and in the airways. Interestingly, we also observed that SARS-CoV-2 infection upregulated *Calca* and *Calcb,* the genes that encodes CGRP-α and -β, but not *Tac1,* the gene that encodes Substance P (Supplemental Fig. 4c). Taken together, these data show that SARS-CoV-2, in the absence of infected airway resident cells, both promotes the transcriptional upregulation and secretion of the immunomodulatory neuropeptide CGRP.

Pain is a hallmark of acute COVID-19 infection and animal models of SARS-CoV-2 infection develop mechanical allodynia both during and after infection.[1,43,46,49] Canonical algogens induce mechanical or thermal sensitization via TRPV1 and TRPA1. [7,50] Thus, we tested if, in addition to acute pain behaviors (Supplemental Fig. 2a, b), PLpro could also drive pain hypersensitivity. We found that PLpro promotes mechanical hypersensitivity following intradermal injection into the hindpaw. PLpro significantly reduced the von Frey paw withdrawal threshold three hours after injection, unlike vehicle injection (Fig. 6a). This PLpro-dependent mechanical sensitization recovered by 24 hours (Fig. 6a). Unlike wild-type and TRPV1-deficient mice, TRPA1-deficient mice did not develop PLpro-induced mechanical sensitization (Fig. 6b). Interestingly, we observed no difference in the thermal sensitivity between vehicle-and PLpro-injected paws, consistent with TRPA1's role in mediating mechanical, but not thermal sensitization (Fig. 6c).[7,51] We next asked whether TRPA1 or TRPV1 are also required for PLpro-induced sneezing. PLpro triggers robust sneezing in wild-type and TRPV1-deficient mice, but not in TRPA1-deficient mice that display little sneezing, comparable to vehicle treatment (Fig, 6d). Altogether these data demonstrate that PLpro activates nociceptors via TRPA1 to drive sneezing and pain behaviors. While TRPA1 is well known to mediate pain and cough,[7,8] we now also demonstrate a role for TRPA1 in viral-associated sneezing and pain.

Symptoms of viral infection such as sneezing, coughing, and headache are consequences of aberrant activation of somatosensory and vagal neurons, that under healthy conditions, regulate breathing, promote protective airway reflexes, and initiate pain sensations. Viral infection has thus far been proposed to drive these symptoms via the release of inflammatory mediators that in turn activate neurons. Here, we demonstrate a new mechanism, by which SARS-CoV-2 directly activates sensory neurons via PLpro to drive sneezing and pain. We show that a single administration of PLpro, in the absence of viral infection or inflammation, is sufficient to activate neurons to drive these behaviors. PLpro activates nociceptors from the trigeminal, dorsal root, and nodose/jugular ganglia innervate the skin and viscera and thus may drive other symptoms of infection such as headache or gastrointestinal

bioRxiv preprint doi: https://doi.org/10.1101/2024.01.10.575114; this version posted January 11, 2024. The copyright holder for this preprint (which was not certified by peer review) is the author/funder, who has granted bioRxiv a license to display the preprint in perpetuity. It is made available under aCC-BY 4.0 International license.

pain. Viral proteases are of great interest as therapeutic targets to inhibit viral replication,[52] and these data suggest that targeting PLpro may also serve to alleviate symptoms associated with COVID-19.

More than 10% of SARS-CoV-2 patients experience chronic symptoms well after the acute infection phase, known as Long COVID.[1,53] The most prevalent Long COVID symptoms include headache (40% of people who experience Long COVID), shortness of breath, (37%), persistent cough (27%), sore throat (27%), and chest pain (23%). Though less common, peripheral neuropathy symptoms (pins and needles and numbness, 2%) have also been reported at a greater frequency than in control groups.[49] But, the mechanisms that drive these long-lasting changes, particularly in the nervous system, remain unclear. A hallmark of neuropathic diseases like chronic pain and itch are transcriptional changes in the sensory ganglia that drive aberrant neuronal activity.[54–56] Long term alterations in nociceptor function are a critical starting point to understanding the mechanisms that drive Long COVID. Future studies are needed to assess whether infection-induced gene expression changes persist beyond infection, and whether PLpro, like other viral proteins, lingers in the body after active infection to contribute to Long COVID symptoms.[57,58]

bioRxiv preprint doi: https://doi.org/10.1101/2024.01.10.575114; this version posted January 11, 2024. The copyright holder for this preprint (which was not certified by peer review) is the author/funder, who has granted bioRxiv a license to display the preprint in perpetuity. It is made available under aCC-BY 4.0 International license.

Methods

Mouse studies

All mice were housed in standard conditions in accordance with standards approved by the Animal Care and Use Committee of the University of California Berkeley (14-hour light: 10-hour dark cycle, 21 °C). Wild-type C57BL/6N mice were obtained from Charles River and C57BL/6J were obtained from Jackson Laboratories and raised in-house. All experiments were performed under the policies and recommendations of the International Association for the Study of Pain and approved by the University of California Berkeley Animal Care and Use Committee. Where appropriate, genotyping was performed by Transnetyx using real-time PCR. Mouse lines used in this study included Pirt-Cre (Pirttm3.1(cre)Xzd) gift from Dr Xinzhong Dong (Johns Hopkins University, Baltimore, MD) and Ai96 (B6J.Cg-Gt(ROSA)26Sortm96$^{(CAG-GCaMP6s)Hze}$/MwarJ), Trpv1 KO; Trpv1tm1Jul RRID:MGI:4417977, and Trpa1 / (A1 KO; Trpa1tm1Jul, RRID:MGI:3696956) from Jackson Laboratories.

Calcium imaging

In vivo calcium imaging of trigeminal ganglion

In vivo calcium imaging of trigeminal neurons was conducted in anesthetized mice as previously described.[59] In brief, Pirt-Cre;GCaMP6s mice were anesthetized with ketamine and xylazine (100 and 10 mg/kg intraperitoneal) and body temperature was maintained at 36°C throughout surgery and imaging. Mice were head-fixed on a custom chamber and tracheotomized to allow normal breathing and restrict intranasal perfusion to the upper airways. A unilateral craniotomy and hemispherectomy was performed to expose the dorsal surface of the right trigeminal ganglion. Hemostasis was achieved using Surgifoam gel sponge (Ethicon 1972). Images were collected at 5 Hz using a Sutter Movable Objective Microscope (MOM) equipped with an Olympus XL-Fluor 4X/340 (NA 0.28) lens, ORCA-Fusion BT Digital CMOS camera. 480nM illumination was provided by Lambda 721 (Sutter). Intranasal perfusion of 10 µL of SARS-CoV-2 PLpro (R&D #E-611), vehicle (50 mM HEPES, 300 mM NaCl, 1 mM TCEP, 10% (v/v) Glycerol, pH 8.0) dilution was matched to that of PLpro, AITC (Sigma) or (E)- Capsaicin (Tocris) was applied evenly between both nostrils using a P20 pipette. Stimuli were applied at 2-minute intervals.

In vivo imaging analysis

Motion correction and source extraction were performed using Suite2p.[60] Suite2p-generated regions of interest (ROIs) were manually inspected and additional ROIs were manually identified. Neuropil was subtracted with a coefficient of 0.7. F_0 is defined as the median fluorescence 150 frames prior to each stimulus treatment and used to calculate F/F_0. Normalized traces were Gaussian filtered (s = 2). Stimulus responders were identified if the peak F/F_0 response during the 2-minute stimulation period was > 10% of F_0. Cells were identified as spontaneously active if their peak fluorescence was 5%

bioRxiv preprint doi: https://doi.org/10.1101/2024.01.10.575114; this version posted January 11, 2024. The copyright holder for this preprint (which was not certified by peer review) is the author/funder, who has granted bioRxiv a license to display the preprint in perpetuity. It is made available under aCC-BY 4.0 International license.

greater than the median fluorescence during the baseline window prior to any stimulus treatment. The percentage of responsive cells were quantified by pooling all cells that respond to any of the applied stimuli.

Neuronal cultures

TG, DRG, and NJG were cultured as previously described.[36,61] In brief, sensory ganglia were dissected and incubated in warmed Collagenase P (Roche) in Hanks calcium-free balanced salt solution (HBSS) followed by incubation in 0.25% trypsin with gentle agitation. Cells were washed, triturated, and plated in Minimum Essential Medium (MEM, Gibco) supplemented with 10% horse serum (v/v), MEM vitamins, penicillin/streptomycin and L-glutamine. Neonatal TG cultures were plated on poly-D-Lysine coated eight-well chambers for calcium imaging. Adult TG, DRG, and NJG were plated on poly-D-Lysine and laminin-coated eight-well chambers for calcium imaging or 96-well plates for infection and ELISA experiments. Age-matched wild-type (C57BL/6N) controls were prepared on the same day for experiments on neurons from knockout mice. Human iPSC-derived neurons were obtained from Axol Bioscience with informed consent obtained from the donor with ethics committee approval. Neurons were differentiated per manufacturer's instructions and used in calcium imaging 21- and 39-days post differentiation (Axol Bioscience, ax0555).

In vitro calcium imaging

Neuronal Ca^{2+} imaging experiments were carried out as previously described.[61] Neuronal cultures were loaded for 45 min at room temperature with 10 µM Fura-2AM supplemented with 0.01% Pluronic F-127 (wt/vol, Life Technologies) in a physiological Ringer's solution containing (in mM) 140 NaCl, 5 KCl, 10 HEPES, 2 CaCl2, 2 MgCl2 and 10 D-(+)-glucose, pH 7.4. Cells were identified as neurons by eliciting depolarization with high potassium Ringer's solution (75 mM) at the end of each experiment. Acquired images were displayed as the ratio of 340 nm / 380 nm. Fura-2 ratios were normalized to baseline ratio. Responding neurons were defined as those having a > 10% increase from baseline ratio. Human iPSC-derived neurons were defined as responding to > 5% increase from baseline ratio. All calcium imaging analyses were performed using custom python scripts. Neurons were stimulated with SARS-CoV-2 PLpro (R&D #E-611), SARS-CoV PLpro (R&D #E-610), MERS-CoV PLpro (R&D #E-609), SARS-CoV-2 PLpro (Cayman #31817) diluted in Ringer's to the appropriate concentration. Vehicle (50 mM HEPES, 300 mM NaCl, 1 mM TCEP, 10% (v/v) Glycerol, pH 8.0) dilution was matched to that of PLpro. Cells were incubated in the TRPA1 antagonist (HC-030031, Tocris #2896) for 15 minutes prior to imaging experiment.

Neuropeptide release

bioRxiv preprint doi: https://doi.org/10.1101/2024.01.10.575114; this version posted January 11, 2024. The copyright holder for this preprint (which was not certified by peer review) is the author/funder, who has granted bioRxiv a license to display the preprint in perpetuity. It is made available under aCC-BY 4.0 International license.

TG from 7-8 week old mice were cultured overnight as described above and supplemented with 50 ng/uL of nerve growth factor. Cultured neurons were incubated with vehicle or PLpro for 15 min in Ringer's solution at room temperature. For ex vivo trachea stimulation, tracheas were dissected and washed in HBSS for 30 min at 37°C. Tracheas were then incubated in DMEM for 30 min at 37° with gentle rotation for baseline measurement and in DMEM with PLpro or vehicle for 1 hour at 37°C with gentle rotation. After incubation, the supernatant (from in vitro experiments) and medium (from ex vivo experiments) was collected and used to quantify the concentration of neuropeptide using an enzyme linked immunosorbent kit (CGRP: Cayman Chemicals #589001, Substance P: Cayman Chemicals #583751) according to the manufacturer's instructions.

<u>SARS-CoV-2 infection</u>

Human airway epithelial cell and primary mouse neuron infection

Human adenocarcinoma lung epithelial (Calu-3) cells (ATCC, HTB-55) were maintained with RPMI (Fisher Scientific) supplemented with fetal bovine serum (FBS), L-glutamine, penicillin/streptomycin. Approximately 5 x 10^5 Calu-3 cells were incubated with SARS-CoV-2 (WA1) in medium (10% heat-inactivated FBS, sodium pyruvate, HEPES, L-glutamine, penicillin/streptomycin) diluted in phenol-red free RPMI to an MOI of 1 for 24 or 48 hours. Mouse TG were cultured as described above and plated in a 96-well plate. TGs (2 from each animal) across 4 animals were pooled and plated across 8 wells. TG cultures were infected with 5 x 10^5 SARS-CoV-2 virions or medium (mock infection) for 48 hours.

Protease activity

Supernatant from SARS-CoV-2 and mock infected Calu-3 cells was combined with the PLpro substrate, Ubiquitin-Rh110 (R&D Systems, #U-555) at 670 nM in phenol-free RPMI. Fluorescence was measured at 535nM on a plate reader. Samples were run in duplicates. A standard curve was generated by measuring protease activity from recombinant SARS-CoV-2 PLpro (R&D) diluted in mock infection medium at 1, 10, 100, 500, and 1000nM and run on the same plate as experimental samples.

RNA sequencing and analysis

After removing supernatant from infected cells, RNA was isolated with DNA/RNA Shield (Zymo Research) according to the manufacturer's instructions. RNA library preparation (lnc-RNA library with rRNA removal) and sequencing on an Illumina NovaSeq 6000 were performed by Novogene. For quantification of viral transcripts, reads were mapped to the appropriate host (human or mouse) transcriptome concatenated with the SARS-CoV-2 transcriptome using *Kallisto (0.44.0)*. For analysis of infected mouse TG, reads were mapped to the mouse genome concatenated with the SARS-CoV-2 genome using *STAR (2.7.10a)*. Gene counts were extracted using featureCounts in *Subread (2.0.1).*

bioRxiv preprint doi: https://doi.org/10.1101/2024.01.10.575114; this version posted January 11, 2024. The copyright holder for this preprint (which was not certified by peer review) is the author/funder, who has granted bioRxiv a license to display the preprint in perpetuity. It is made available under aCC-BY 4.0 International license.

All but one sample contained more than 80% mapped features; one sample contained 68% mapped reads and was excluded based on the robust principal component analysis method.[62] The uniquely aligned features were used for differential expression analysis using *DESeq2* (*1.36.0)* and the genes were determined to be significantly differentially expressed if they had a base mean > 152, $\log_2$ fold change > 0.37, and adjusted p-value < 0.001. Gene sets associated with functional neuronal subtypes were generated from a previous study.[63] We pooled the same Gene Ontology terms Mouse Genome Informatics (MGI) (updated as of November 2023) except for the itch gene list, which we used from the paper. We tested if these custom gene sets were significantly differentially expressed above chance. In brief, we established a null distribution by sampling random sets of genes (of the same size) from all transcripts expressed across our samples 100,000 times and compared the median absolute $\log_2$ fold change for each custom gene set to the null distribution to determine the empirical p-value.

Behavioral studies

Intranasal behavior and scoring

For intranasal behavioral experiments, mice were acclimated in a custom clear acrylic behavioral chamber (4” x 2” x 6”) with a filter paper bottom (VWR #28321-113) for 10 minutes prior to intranasal treatment. Mice were treated intranasally with vehicle or PLpro with 10 mg/mL Evan’s blue dye in phosphate-buffered solution (PBS) administered 5 μL/nostril (10 uL total) with a P20 pipette. Mice were then placed back in the chamber for another 10 minutes of recording. A GoPro Hero 11 (recording at 2.7 megapixels, 240Hz) was used to record video and ambient audio. An audio recorder (aTTo digital, 16 kHz) placed inside the chambers. Experimenters were blinded to genotype or treatment. Behavior was scored for 2 minutes by individuals blinded to both genotype and treatment. A nose rub was defined as a singular elliptical stroke made by both forepaws simultaneously, directed to the nose. A face wipe was also a singular elliptical stroke of the forelimbs but contacted the face above the nose and below the ears, making a circular motion. The time of each individual nose rub and face wipe was recorded. A bout of body licking was defined as an uninterrupted series of licking the flank, chest, stomach, and tail. The filter paper that was on the bottom of the chamber was scanned and the mean intensity was quantified using *Fiji*, and the mean intensity was inverted (subtracted by 255) and scaled (by 10^8).

Sneeze analysis

Sneeze events were identified from peaks in the audio recordings. Audio recordings were cropped to the start of the experiment and a 2-3 second recording of background sound from the recording was used to denoise in Audacity (Noise reduction: 30 dB, sensitivity: 6, frequency of smoothing: 6 bands). Denoised audio recordings were then analyzed using a custom python script. Audio peaks were

bioRxiv preprint doi: https://doi.org/10.1101/2024.01.10.575114; this version posted January 11, 2024. The copyright holder for this preprint (which was not certified by peer review) is the author/funder, who has granted bioRxiv a license to display the preprint in perpetuity. It is made available under aCC-BY 4.0 International license.

identified using the SciPy "find_peaks" function (peak prominence > 1000 and a minimum distance of 0.2s between peaks). The audio recordings from inside the chamber were aligned to the video and audio recordings from the GoPro (outside the chamber). Audio peaks recorded both inside and outside the chamber were excluded as background noise. Audio peaks that correlated with other behaviors such as biting the chamber and body licking were excluded.

Cheek behavior

Itch and acute pain behavioral measurements were performed as described previously.[61] In brief, mice were shaved and singly housed one week prior to the experiment. Mice were acclimated to the chamber for an hour to the chamber both the day prior and before injection. Compounds were injected intradermally (20 µL) into the cheek. Behavior was videotaped for one hour and scored for either the first 30 min (scratching) or the first 5 min (wiping) as previously described.[64]

Von Frey and radiant heat behavior

Mice were lightly anesthetized with isoflurane (2.5%) and PLpro or vehicle was injected intradermally into the plantar surface of the hind paw (15 µL). For von Frey, both hind paws were injected, one with treatment and the other with vehicle solution. For radiant heat, only the left hind paw was injected with either treatment or vehicle solution. Mice were acclimated in behavioral chambers for 2 subsequent days for at least an hour followed by an additional 30 minutes of acclimation with the investigator in the room. Mechanical threshold was measured using calibrated von Frey monofilaments (Touch Test) on a metal grate platform (IITC). Von Frey was performed as previously described[65,66] using the up-down method[67]. Measurements were taken one day prior to injection, the 3-, 27-, and 51-hours following injection. Radiant heat assay was performed using the Hargreaves test system (IITC Life Science) as previously described.[65,68] A glass platform was set at 30°C during the acclimation and throughout the experiment and the radiant heat source raised the platform temperature to 41.5°C within 5s and to 44.9°C within 10s, as measured by a fast temperature probe (Physitemp). Valid responses for both von Frey and radiant heat included fast paw withdrawal, licking/biting/shaking of the affected paw, or flinching. Experimenter was blind to treatment and genotype.

Statistical reporting

Unless otherwise noted, data are expressed as mean ± standard error of the mean (SEM). All performed statistical tests were two-tailed using Prism. Schematics were made using Biorender. RNAseq analysis was performed using R. All other data were analyzed using Python.

Data Availability

bioRxiv preprint doi: https://doi.org/10.1101/2024.01.10.575114; this version posted January 11, 2024. The copyright holder for this preprint (which was not certified by peer review) is the author/funder, who has granted bioRxiv a license to display the preprint in perpetuity. It is made available under aCC-BY 4.0 International license.

RNA sequencing data were deposited into the Gene Expression Omnibus database under accession number GSE252056.

Acknowledgements:

We are grateful Drs. Sven-Eric Jordt and Anabel De Caceres Bustos and Christopher Cook, Odilia Liu, and Abigail Mende for critical discussions and pilot experiments, Drs. Rachel Brem, Jay Parrish and the MBL Neurobiology course 2022 students for help with pilot RNAseq analysis, and Dr. Olivia Goldman and Erin Aisenberg for critical review of the manuscript. S.S.M. funded by a predoctoral fellowship from the National Science Foundation to S.S.M. (NSF GRFP DGE1752814). This work was funded by grants from the National Institutes of Health to D.M.B (OD TR01 NS116992) and the Howard Hughes Medical Institute.

Author contributions:

S.S.M. and D.M.B conceived the project and designed experiments. S.S.M led and performed experiments, analyzed data, and prepared figures. R.S. and C.V. performed paw and cheek behavior experiments. U.V., R.S., and S.S.M. performed intranasal behavior scoring. M.C. performed and J.S.C. supervised infection experiments. Z.G. performed RNAseq analysis. Y.M. performed pilot and provided technical assistance for in vivo imaging experiments. S.S.M. and D.M.B wrote the manuscript with input from all authors.

bioRxiv preprint doi: https://doi.org/10.1101/2024.01.10.575114; this version posted January 11, 2024. The copyright holder for this preprint (which was not certified by peer review) is the author/funder, who has granted bioRxiv a license to display the preprint in perpetuity. It is made available under aCC-BY 4.0 International license.

References

1. Bliddal, S. *et al.* Acute and persistent symptoms in non-hospitalized PCR-confirmed COVID-19 patients. *Sci. Rep.* **11**, 13153 (2021).
2. Li, F. *et al.* Sneezing reflex is mediated by a peptidergic pathway from nose to brainstem. *Cell* **184**, 3762–3773.e10 (2021).
3. Finger, T. E., St Jeor, V. L., Kinnamon, J. C. & Silver, W. L. Ultrastructure of substance P- and CGRP-immunoreactive nerve fibers in the nasal epithelium of rodents. *J. Comp. Neurol.* **294**, 293–305 (1990).
4. Baral, P. *et al.* Nociceptor sensory neurons suppress neutrophil and γδ T cell responses in bacterial lung infections and lethal pneumonia. *Nat. Med.* **24**, 417–426 (2018).
5. Pinho-Ribeiro, F. A., Verri, W. A., Jr & Chiu, I. M. Nociceptor Sensory Neuron-Immune Interactions in Pain and Inflammation. *Trends Immunol.* **38**, 5–19 (2017).
6. Prescott, S. L., Umans, B. D., Williams, E. K., Brust, R. D. & Liberles, S. D. An Airway Protection Program Revealed by Sweeping Genetic Control of Vagal Afferents. *Cell* **181**, 574–589.e14 (2020).
7. Bautista, D. M. *et al.* TRPA1 mediates the inflammatory actions of environmental irritants and proalgesic agents. *Cell* **124**, 1269–1282 (2006).
8. Grace, M. S. & Belvisi, M. G. TRPA1 receptors in cough. *Pulm. Pharmacol. Ther.* **24**, 286–288 (2011).
9. Caceres, A. I. *et al.* A sensory neuronal ion channel essential for airway inflammation and hyperreactivity in asthma. *Proc. Natl. Acad. Sci. U. S. A.* **106**, 9099–9104 (2009).
10. Grant, M. C. *et al.* The prevalence of symptoms in 24,410 adults infected by the novel coronavirus (SARS-CoV-2; COVID-19): A systematic review and meta-analysis of 148 studies from 9 countries. *PLoS One* **15**, e0234765 (2020).
11. Prescott, S. L. & Liberles, S. D. Internal senses of the vagus nerve. *Neuron* **110**, 579–599 (2022).
12. Bin, N.-R. *et al.* An airway-to-brain sensory pathway mediates influenza-induced sickness. *Nature* **615**, 660–667 (2023).

bioRxiv preprint doi: https://doi.org/10.1101/2024.01.10.575114; this version posted January 11, 2024. The copyright holder for this preprint (which was not certified by peer review) is the author/funder, who has granted bioRxiv a license to display the preprint in perpetuity. It is made available under aCC-BY 4.0 International license.

13. Barragán-Iglesias, P. *et al.* Type I Interferons Act Directly on Nociceptors to Produce Pain Sensitization: Implications for Viral Infection-Induced Pain. *J. Neurosci.* **40**, 3517–3532 (2020).
14. McFarland, A. J., Yousuf, M. S., Shiers, S. & Price, T. J. Neurobiology of SARS-CoV-2 interactions with the peripheral nervous system: implications for COVID-19 and pain. *Pain Rep* **6**, e885 (2021).
15. Patil, M. J. *et al.* Acute activation of bronchopulmonary vagal nociceptors by type I interferons. *J. Physiol.* **598**, 5541–5554 (2020).
16. Staurengo-Ferrari, L., Deng, L. & Chiu, I. M. Interactions between nociceptor sensory neurons and microbial pathogens in pain. *Pain* **163**, S57–S68 (2022).
17. Akira, S., Uematsu, S. & Takeuchi, O. Pathogen recognition and innate immunity. *Cell* **124**, 783–801 (2006).
18. Reddy, V. B., Iuga, A. O., Shimada, S. G., LaMotte, R. H. & Lerner, E. A. Cowhage-evoked itch is mediated by a novel cysteine protease: a ligand of protease-activated receptors. *J. Neurosci.* **28**, 4331–4335 (2008).
19. Serhan, N. *et al.* House dust mites activate nociceptor-mast cell clusters to drive type 2 skin inflammation. *Nat. Immunol.* **20**, 1435–1443 (2019).
20. Perner, C. *et al.* Substance P Release by Sensory Neurons Triggers Dendritic Cell Migration and Initiates the Type-2 Immune Response to Allergens. *Immunity* **53**, 1063–1077.e7 (2020).
21. Hassler, S. N. *et al.* The cellular basis of protease-activated receptor 2-evoked mechanical and affective pain. *JCI Insight* **5**, (2020).
22. Pradhananga, S., Tashtush, A. A., Allen-Vercoe, E., Petrof, E. O. & Lomax, A. E. Protease-dependent excitation of nodose ganglion neurons by commensal gut bacteria. *J. Physiol.* **598**, 2137–2151 (2020).
23. Gu, Q. & Lee, L.-Y. House dust mite potentiates capsaicin-evoked Ca2+ transients in mouse pulmonary sensory neurons via activation of protease-activated receptor-2. *Exp. Physiol.* **97**, 534–543 (2012).
24. Jimenez-Vargas, N. N. *et al.* Protease-activated receptor-2 in endosomes signals persistent pain

bioRxiv preprint doi: https://doi.org/10.1101/2024.01.10.575114; this version posted January 11, 2024. The copyright holder for this preprint (which was not certified by peer review) is the author/funder, who has granted bioRxiv a license to display the preprint in perpetuity. It is made available under aCC-BY 4.0 International license.

of irritable bowel syndrome. *Proc. Natl. Acad. Sci. U. S. A.* **115**, E7438–E7447 (2018).

25. Deng, L. *et al. Staphylococcus aureus* Drives Itch and Scratch-Induced Skin Damage Through a V8 Protease-PAR1 Axis. (2023) doi:10.2139/ssrn.4318823.
26. Kwong, K., Nassenstein, C., de Garavilla, L., Meeker, S. & Undem, B. J. Thrombin and trypsin directly activate vagal C-fibres in mouse lung via protease-activated receptor-1. *J. Physiol.* **588**, 1171–1177 (2010).
27. Liu, Q. *et al.* The distinct roles of two GPCRs, MrgprC11 and PAR2, in itch and hyperalgesia. *Sci. Signal.* **4**, ra45 (2011).
28. Shin, D. *et al.* Papain-like protease regulates SARS-CoV-2 viral spread and innate immunity. *Nature* **587**, 657–662 (2020).
29. Hartenian, E. *et al.* The molecular virology of Coronaviruses. *J. Biol. Chem.* (2020) doi:10.1074/jbc.REV120.013930.
30. Baraniuk, J. N. & Kim, D. Nasonasal reflexes, the nasal cycle, and sneeze. *Curr. Allergy Asthma Rep.* **7**, 105–111 (2007).
31. Hargreaves, K. M. Orofacial pain. *Pain* **152**, S25–S32 (2011).
32. Iwasaki, N. *et al.* Allergen endotoxins induce T-cell-dependent and non-IgE-mediated nasal hypersensitivity in mice. *J. Allergy Clin. Immunol.* **139**, 258–268.e10 (2017).
33. Kayasuga, R., Sugimoto, Y., Watanabe, T. & Kamei, C. Histamine H1 receptors are involved in mouse nasal allergic responses: a demonstration with H1 receptor-deficient mice. *Int. Immunopharmacol.* **2**, 745–750 (2002).
34. LaMotte, R. H., Shimada, S. G. & Sikand, P. Mouse models of acute, chemical itch and pain in humans. *Exp. Dermatol.* **20**, 778–782 (2011).
35. Grace, M., Birrell, M. A., Dubuis, E., Maher, S. A. & Belvisi, M. G. Transient receptor potential channels mediate the tussive response to prostaglandin E2 and bradykinin. *Thorax* **67**, 891–900 (2012).
36. Hill, R. Z., Morita, T., Brem, R. B. & Bautista, D. M. S1PR3 Mediates Itch and Pain via Distinct TRP Channel-Dependent Pathways. *J. Neurosci.* **38**, 7833–7843 (2018).

bioRxiv preprint doi: https://doi.org/10.1101/2024.01.10.575114; this version posted January 11, 2024. The copyright holder for this preprint (which was not certified by peer review) is the author/funder, who has granted bioRxiv a license to display the preprint in perpetuity. It is made available under aCC-BY 4.0 International license.

37. Chiu, I. M. *et al.* Bacteria activate sensory neurons that modulate pain and inflammation. *Nature* **501**, 52–57 (2013).

38. Wang, H. & Woolf, C. J. Pain TRPs. *Neuron* **46**, 9–12 (2005).

39. Sun, H., Meeker, S. & Undem, B. J. Role of TRP channels in Gq-coupled protease-activated receptor 1-mediated activation of mouse nodose pulmonary C-fibers. *Am. J. Physiol. Lung Cell. Mol. Physiol.* **318**, L192–L199 (2020).

40. Báez-Santos, Y. M., Mielech, A. M., Deng, X., Baker, S. & Mesecar, A. D. Catalytic function and substrate specificity of the papain-like protease domain of nsp3 from the Middle East respiratory syndrome coronavirus. *J. Virol.* **88**, 12511–12527 (2014).

41. Osipiuk, J. *et al.* Structure of papain-like protease from SARS-CoV-2 and its complexes with non-covalent inhibitors. *Nat. Commun.* **12**, 743 (2021).

42. Meinhardt, J. *et al.* Olfactory transmucosal SARS-CoV-2 invasion as a port of central nervous system entry in individuals with COVID-19. *Nat. Neurosci.* (2020) doi:10.1038/s41593-020-00758-5.

43. Joyce, J. D. *et al.* SARS-CoV-2 Infects Peripheral and Central Neurons Before Viremia, Facilitated by Neuropilin-1. *bioRxiv* 2022.05.20.492834 (2023) doi:10.1101/2022.05.20.492834.

44. Flamier, A., Bisht, P., Richards, A., Tomasello, D. L. & Jaenisch, R. Human iPS cell-derived sensory neurons can be infected by SARS-CoV-2. *iScience* **26**, 107690 (2023).

45. Abdullah, H., Heaney, L. G., Cosby, S. L. & McGarvey, L. P. A. Rhinovirus upregulates transient receptor potential channels in a human neuronal cell line: implications for respiratory virus-induced cough reflex sensitivity. *Thorax* **69**, 46–54 (2014).

46. Serafini, R. A. *et al.* SARS-CoV-2 airway infection results in the development of somatosensory abnormalities in a hamster model. *Sci. Signal.* **16**, eade4984 (2023).

47. Basbaum, A. I., Bautista, D. M., Scherrer, G. & Julius, D. Cellular and molecular mechanisms of pain. *Cell* **139**, 267–284 (2009).

48. Tamari, M. *et al.* Sensory neurons promote immune homeostasis in the lung. *Cell* **187**, 44–61.e17 (2024).

bioRxiv preprint doi: https://doi.org/10.1101/2024.01.10.575114; this version posted January 11, 2024. The copyright holder for this preprint (which was not certified by peer review) is the author/funder, who has granted bioRxiv a license to display the preprint in perpetuity. It is made available under aCC-BY 4.0 International license.

49. Sudre, C. H. *et al.* Attributes and predictors of long COVID. *Nat. Med.* **27**, 626–631 (2021).

50. Caterina, M. J. *et al.* Impaired nociception and pain sensation in mice lacking the capsaicin receptor. *Science* **288**, 306–313 (2000).

51. Lennertz, R. C., Kossyreva, E. A., Smith, A. K. & Stucky, C. L. TRPA1 mediates mechanical sensitization in nociceptors during inflammation. *PLoS One* **7**, e43597 (2012).

52. Majerová, T. & Konvalinka, J. Viral proteases as therapeutic targets. *Mol. Aspects Med.* **88**, 101159 (2022).

53. Davis, H. E., McCorkell, L., Vogel, J. M. & Topol, E. J. Long COVID: major findings, mechanisms and recommendations. *Nat. Rev. Microbiol.* **21**, 133–146 (2023).

54. Walsh, C. M. *et al.* Neutrophils promote CXCR3-dependent itch in the development of atopic dermatitis. *Elife* **8**, (2019).

55. North, R. Y. *et al.* Electrophysiological and transcriptomic correlates of neuropathic pain in human dorsal root ganglion neurons. *Brain* **142**, 1215–1226 (2019).

56. Renthal, W. *et al.* Transcriptional Reprogramming of Distinct Peripheral Sensory Neuron Subtypes after Axonal Injury. *Neuron* **108**, 128–144.e9 (2020).

57. Klein, J. *et al.* Distinguishing features of long COVID identified through immune profiling. *Nature* **623**, 139–148 (2023).

58. Chen, B., Julg, B., Mohandas, S., Bradfute, S. B. & RECOVER Mechanistic Pathways Task Force. Viral persistence, reactivation, and mechanisms of long COVID. *Elife* **12**, (2023).

59. Moayedi, Y. *et al.* The cellular basis of mechanosensation in mammalian tongue. *Cell Rep.* **42**, 112087 (2023).

60. Pachitariu, M. *et al.* Suite2p: beyond 10,000 neurons with standard two-photon microscopy. *bioRxiv* 061507 (2017) doi:10.1101/061507.

61. Wilson, S. R. *et al.* TRPA1 is required for histamine-independent, Mas-related G protein-coupled receptor-mediated itch. *Nat. Neurosci.* **14**, 595–602 (2011).

62. Chen, X., Zhang, B., Wang, T., Bonni, A. & Zhao, G. Robust principal component analysis for accurate outlier sample detection in RNA-Seq data. *BMC Bioinformatics* **21**, 269 (2020).

bioRxiv preprint doi: https://doi.org/10.1101/2024.01.10.575114; this version posted January 11, 2024. The copyright holder for this preprint (which was not certified by peer review) is the author/funder, who has granted bioRxiv a license to display the preprint in perpetuity. It is made available under aCC-BY 4.0 International license.

63. Usoskin, D. *et al.* Unbiased classification of sensory neuron types by large-scale single-cell RNA sequencing. *Nat. Neurosci.* **18**, 145–153 (2015).

64. Shimada, S. G. & LaMotte, R. H. Behavioral differentiation between itch and pain in mouse. *Pain* **139**, 681–687 (2008).

65. Tsunozaki, M. *et al.* A ‘toothache tree’ alkylamide inhibits Aδ mechanonociceptors to alleviate mechanical pain. *J. Physiol.* **591**, 3325–3340 (2013).

66. Chaplan, S. R., Bach, F. W., Pogrel, J. W., Chung, J. M. & Yaksh, T. L. Quantitative assessment of tactile allodynia in the rat paw. *J. Neurosci. Methods* **53**, 55–63 (1994).

67. Dixon, W. J. The Up-and-Down Method for Small Samples. *J. Am. Stat. Assoc.* **60**, 967–978 (1965).

68. Hargreaves, K., Dubner, R., Brown, F., Flores, C. & Joris, J. A new and sensitive method for measuring thermal nociception in cutaneous hyperalgesia. *Pain* **32**, 77–88 (1988).

bioRxiv preprint doi: https://doi.org/10.1101/2024.01.10.575114; this version posted January 11, 2024. The copyright holder for this preprint (which was not certified by peer review) is the author/funder, who has granted bioRxiv a license to display the preprint in perpetuity. It is made available under aCC-BY 4.0 International license.

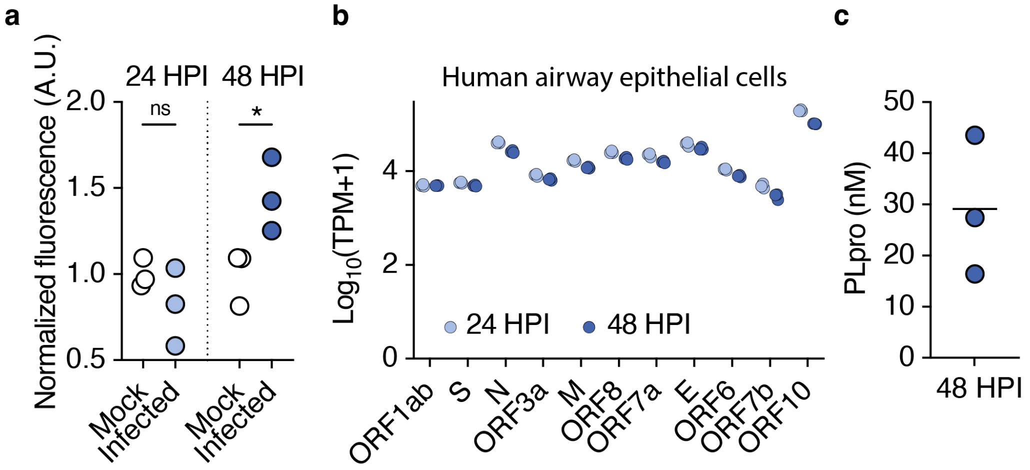

Figure 1. SARS-CoV-2 PLpro is released from infected airway epithelial cells.
a, PLpro proteolytic activity from the supernatant of SARS-CoV-2 or mock infected Calu-3 cells 24 and 48 HPI, measured by the fluorescence of cleaved substrate, normalized across samples from the same day. One-way ANOVA: p=0.014, F(3,8)=6.790; Holm-Šídák's multiple comparisons, $p_{\text{mock vs. infected 24h}}$ =0.244, $p_{\text{mock vs. infected 48h}}$ =0.030, *n*=3 wells per group. **b,** SARS-CoV-2 transcripts from Calu-3 cells infected with SARS-CoV-2 (multiplicity of infection = 1) for 24 and 48 hours post infection (HPI), transcripts per million (TPM), *n*=3 wells. **c**, Interpolated concentration of PLpro in the supernatant of Calu-3 cells 48 hours post infection, line represents mean.

bioRxiv preprint doi: https://doi.org/10.1101/2024.01.10.575114; this version posted January 11, 2024. The copyright holder for this preprint (which was not certified by peer review) is the author/funder, who has granted bioRxiv a license to display the preprint in perpetuity. It is made available under aCC-BY 4.0 International license.

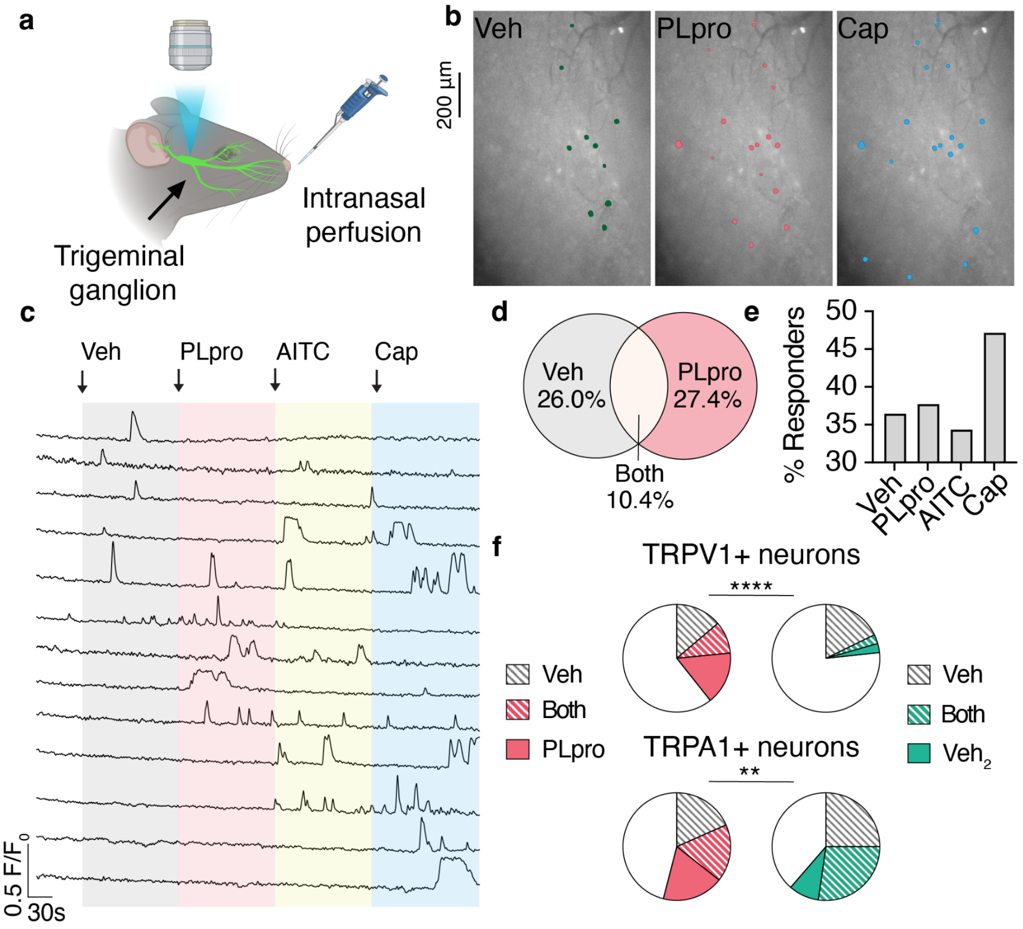

Figure 2. PLpro activates upper airway-innervating neurons in vivo.
a, Schematic of in vivo imaging preparation. **b,** Image of trigeminal ganglion overlaid with the regions of interest of neurons that respond to intranasal perfusion (10 µL) of vehicle (Veh), 10 µM PLpro, and 100 µM capsaicin (Cap). **c,** Representative calcium transients in response to intranasal perfusion of Veh, 10 µM PLpro, 1 mM AITC, 100 µM Cap. **d,** Venn diagram of neurons responsive to vehicle (112/307) and PLpro (116/307), *n*=307 neurons from 6 mice. PLpro activates a subset of vehicle-sensitive neurons (32/112). **e,** Percent of recorded neurons that respond to each stimulus. **f,** Vehicle and PLpro activate a subset of TRPV1+ (capsaicin-responsive) neurons and TRPA1+ (AITC-responsive) neurons. Top Left, percent of TRPV1+ neurons responsive to vehicle only: 13.8%, both vehicle and PLpro: 9.7%, PLpro only: 15.9%, *n*=145 neurons from 6 mice; Top Right, percent of TRPV1+ neurons responsive to the first delivery of vehicle only: 17.8%, both first and second (Veh_2) deliveries of vehicle: 2.7%, second delivery of vehicle only: 2.7%, *n*=73 neurons from 3 mice, Chi-square test χ^2 = 112.5, df = 3, p<0.0001. Bottom Left, percent of TRPA1+ neurons responsive to vehicle only: 18.4%, both vehicle and PLpro: 17.2%, PLpro only: 18.4%, *n*=87 neurons from 4 mice; Bottom Right, percent of TRPA1+ neurons responsive to the first delivery of vehicle only: 25.0%, both deliveries of vehicle: 27.3%, second delivery of vehicle only: 9.1%, *n*=44 neurons from 3 mice, Chi-square test χ^2 = 14.2, df = 3, p = 0.003.

bioRxiv preprint doi: https://doi.org/10.1101/2024.01.10.575114; this version posted January 11, 2024. The copyright holder for this preprint (which was not certified by peer review) is the author/funder, who has granted bioRxiv a license to display the preprint in perpetuity. It is made available under aCC-BY 4.0 International license.

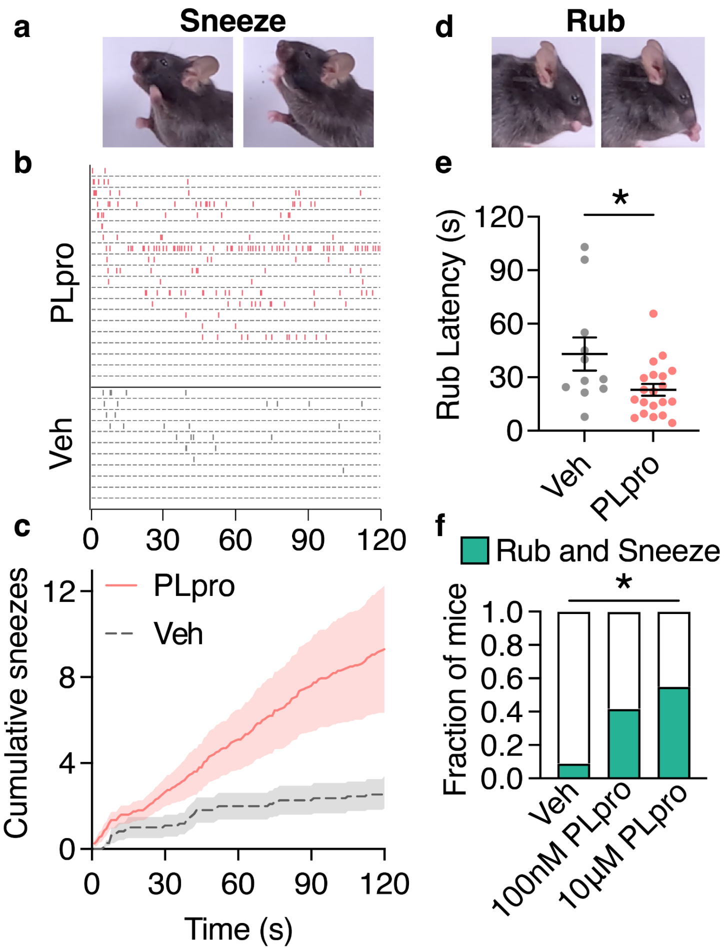

Figure 3. PLpro elicits sneezes and nose rubs in mice.
a, Sequential images of a sneeze following intranasal treatment of 10 µM PLpro (10 µL). **b,** Raster plot of sneezes from individual mice treated with 10 µM PLpro or vehicle identified from audio recordings. **c,** Average cumulative sneeze count of 10 µM PLpro and vehicle treated mice over 2 minutes **d,** Sequential images of a nose rub following intranasal treatment **e,** 10 µM PLpro treated mice display a shorter latency to the first nose rub than vehicle treated mice, t-test: t=2.469, df=29, p=0.020. **f,** Fraction of mice that elicited a rub and sneeze within the first 30 seconds after treatment. (Vehicle: 9.0%, PLpro (100 nM): 41.7%, PLpro (10 µM): 55.0%). Fisher's exact test ($p_{\text{vehicle vs. 100nM PLpro}}$ =0.252, $p_{\text{vehicle vs. 10µM PLpro}}$ =0.020). For all data in this figure, vehicle (*n*=11), 100 nM PLpro (*n*=12), and 10 µM PLpro (*n*=20). Error bars and shading represent the mean ± standard error of the mean (SEM), *n* = biological replicates (animals).

bioRxiv preprint doi: https://doi.org/10.1101/2024.01.10.575114; this version posted January 11, 2024. The copyright holder for this preprint (which was not certified by peer review) is the author/funder, who has granted bioRxiv a license to display the preprint in perpetuity. It is made available under aCC-BY 4.0 International license.

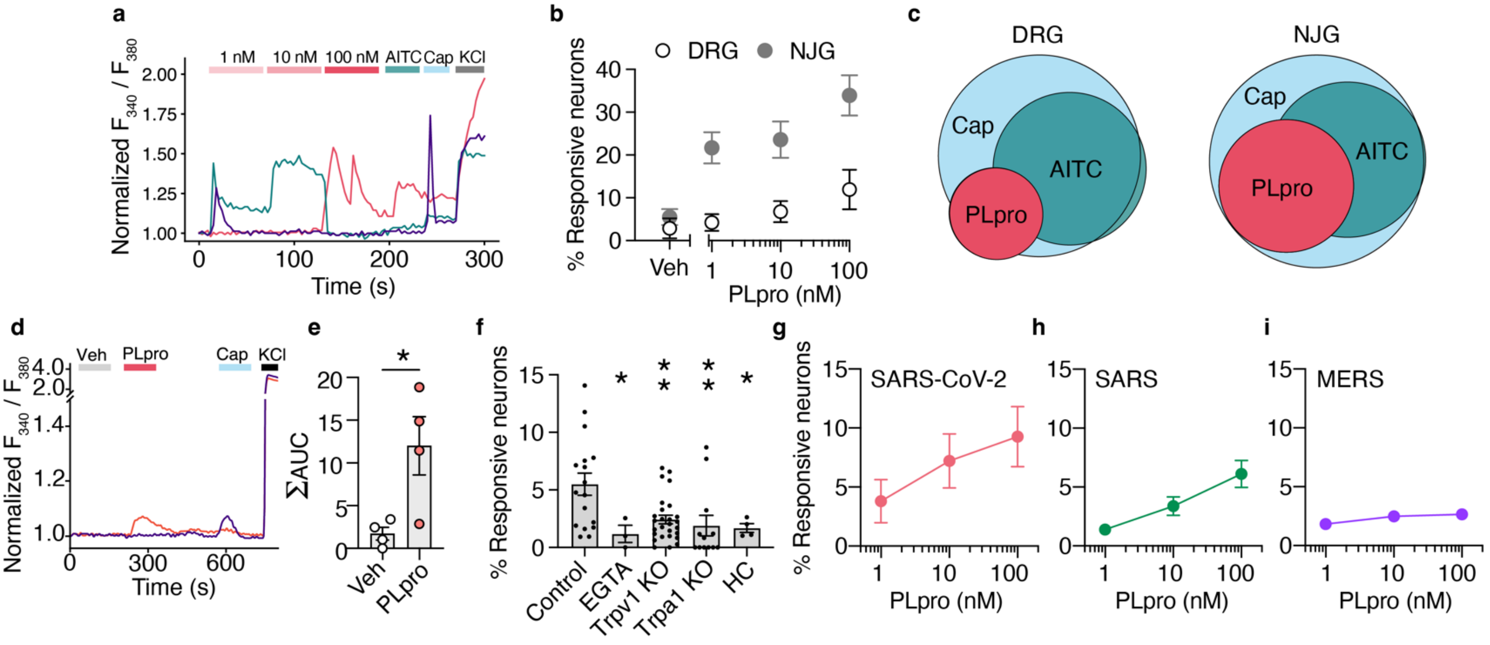

Figure 4. PLpro directly activates nociceptors.
a, Representative calcium transients from PLpro responders from cultured neonatal mouse trigeminal ganglia (TG). **b,** PLpro activates subsets of neurons from adult mouse dorsal root ganglia (DRG; Veh: 2.9%, 1 nM: 4.2%, 10 nM: 6.8%, 100 nM: 11.9%, *n*=5) and adult mouse nodose and jugular ganglia (NJG; Veh: 5.5%, 1 nM: 21.7%, 10 nM: 23.6%, 100 nM: 33.9%, *n*=8). **c,** Venn diagram of PLpro, AITC, and Cap-responsive neurons in DRG and NJG. Of the neurons that respond to these 3 stimuli, Left, in the DRG, 20% responded to PLpro, an additional 48% responded to AITC, and 32% responded to Cap alone. Right, in the NJG, 38% responded to PLpro, an additional 34% responded to AITC, and 28% responded to Cap alone. **d,** Representative calcium transients evoked by PLpro and capsaicin (Cap) in human iPSC-derived neurons. **e,** PLpro induces significantly more calcium influx (area under the curve, AUC) than vehicle, t-test: t=2.953, df=6, p=0.026, *n*=4 wells each. **f,** Percent of neonatal mouse TG neurons that respond to 250nM PLpro in control neurons from wild-type C57BL/6N mice in physiological extracellular calcium (2 mM Ca^{2+}, 5.5%, *n*=17) is greater than in the absence of extracellular calcium (EGTA, 1.2%, *n*=3) and in neurons from Trpv1 KO (2.4%, *n*=27) and Trpa1 KO (1.9%, *n*=12) mice, and in wild-type mice in the presence of TRPA1 antagonist (HC, 1.7%, *n*=4). One-way ANOVA: p=0.003, F(4,58)=2.803; Holm-Šídák's multiple comparisons, $p_{\text{Control vs. EGTA}}$ =0.035, $p_{\text{Control vs. Trpv1 KO}}$ =0.004, $p_{\text{Control vs. Trpa1 KO}}$ =0.004, $p_{\text{Control vs. EGTA}}$ =0.035. **g-i,** SARS-CoV-2 and SARS, but not MERS PLpro activate subsets of neonatal mouse TG neurons in a dose-dependent manner. **g**, SARS-CoV-2 PLpro: 1nM: 3.8%, 10 nM: 7.2%, 100 nM: 9.3%, *n*=5. **h,** SARS PLpro**:** 1 nM: 1.4%, 10 nM: 3.4%, 100nM: 6.1%, *n*=5. **i,** MERS PLpro: 1nM: 1.8%, 10nM: 2.5%, 100nM: 2.7%, *n*=6. *p <0.05, **p<0.01. Error bars represent the mean ± SEM, *n* indicates replicates (wells).

bioRxiv preprint doi: https://doi.org/10.1101/2024.01.10.575114; this version posted January 11, 2024. The copyright holder for this preprint (which was not certified by peer review) is the author/funder, who has granted bioRxiv a license to display the preprint in perpetuity. It is made available under aCC-BY 4.0 International license.

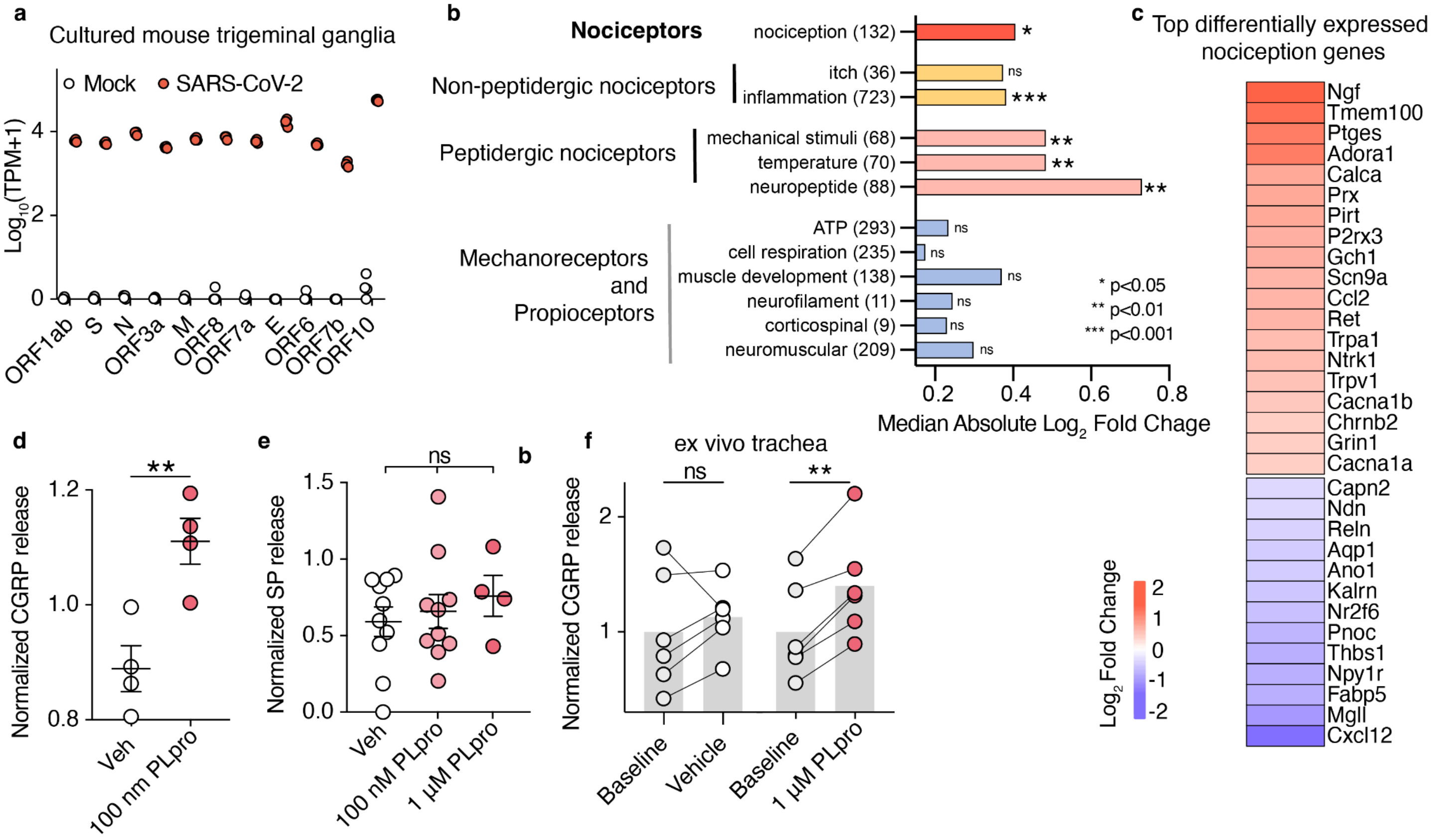

Figure 5. SARS-CoV-2 infection and PLpro activation share a key nociceptive signature.
a, SARS-CoV-2 viral transcript expression 48 hours after infection in cultured adult trigeminal ganglia, mock infected: *n*=4 wells, SARS-CoV-2 infected: *n*=3 wells. **b,** Infection alters gene sets associated with nociception but not mechanoreceptors and proprioceptors. The median absolute $\log_2$ fold change from each custom gene set was tested against 100,000 permutations of randomly selected gene sets of the same size, size of each gene set listed in parenthesis. **c,** Heatmap of the most highly differentially expressed nociception-associated transcripts ($\log_2$ fold change of infection vs mock). **d,** PLpro elicits CGRP release from cultured TG. t-test: t=3.928, df=6, p=0.008, *n* = 4 per group, biological replicates (animal) **e,** PLpro does not elicit the release of Substance P from cultured TG, One-way ANOVA: p=0.673, F(2,21)=0.215, vehicle (*n*=10), 100 nM PLpro (*n*=10), and 1 µM PLpro (*n*=4) replicates (wells from pooled animals) **f,** PLpro but not vehicle increases CGRP release from baseline measurements in an ex vivo trachea preparation. Baseline vs. vehicle: one sample t-test: t=0.909, df=5, p=0.405, baseline vs. 1µM PLpro: one sample t-test: t=6.80, df=5, p=0.001, *n* = 6, biological replicates (animal) per group. *p <0.05, **p<0.01, ***p<0.001. Bar represents mean and error bars represent the mean ± SEM.

bioRxiv preprint doi: https://doi.org/10.1101/2024.01.10.575114; this version posted January 11, 2024. The copyright holder for this preprint (which was not certified by peer review) is the author/funder, who has granted bioRxiv a license to display the preprint in perpetuity. It is made available under aCC-BY 4.0 International license.

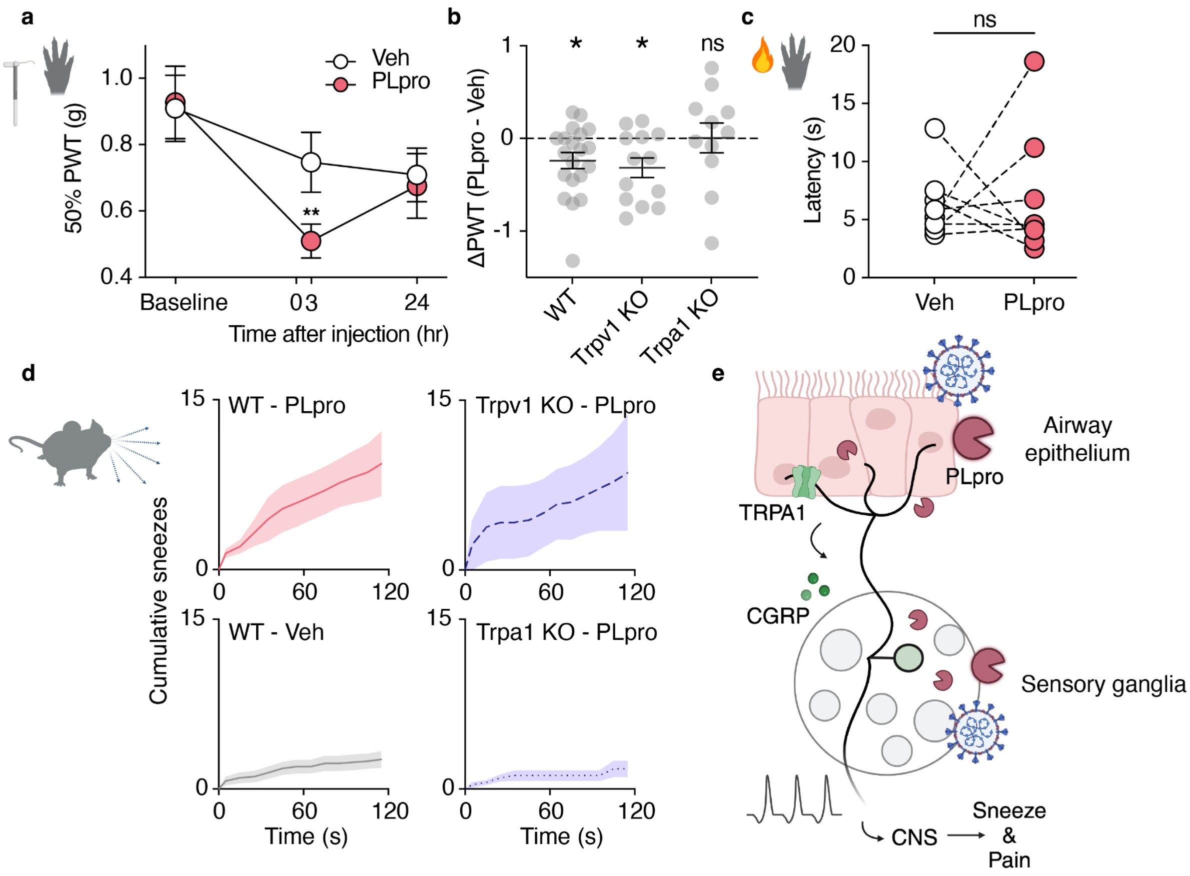

Fig 6. SARS-CoV-2 PLpro-evoked sneeze and pain require TRPA1.
a, 50 μM PLpro induces a reduction in the paw withdrawal threshold (PWT) for force 3, but not 24 hours post injection in PLpro injected paws, but not vehicle injected paws compared to baseline. (24 hours pre-injection). Two-way ANOVA: $p_{time}= 0.008$, $F(1.793, 64.56) = 5.477$; Holm-Šídák's multiple comparisons, Veh: p-adjusted $_{Baseline\ vs.\ 3\ hours}$ =0.361, p-adjusted $_{Baseline\ vs.\ 24\ hours}$ =0.079; PLpro: p-adjusted $_{Baseline\ vs.\ 3\ hours}$ =0.008, p-adjusted $_{Baseline\ vs.\ 24\ hours}$ =0.117, *n*= 19. **b**, Wild-type C57BL/6J (WT) and Trpv1 KO mice, but not Trpa1 KO mice develop mechanical hypersensitivity 3 hours post injection. WT: one sample t-test: t=2.746, df=19, p=0.013, *n* = 11; Trpv1 KO: one sample t-test: t=3.001, df=20, p=0.011, n = 13; Trpa1 KO: one sample t-test: t=0.039, df=10, p=0.970, *n* = 11. **c,** 50 μM PLpro injection does not change the latency to respond to the radiant heat stimulation, paired t-test: t=0.233, df=7, p=0.822, *n* = 8. **d,** Intranasal treatment of 10 μM PLpro triggers more sneezing in Top Left, WT and Top Right, Trpv1 KO mice than Bottom Right, Trpa1 KO mice or Bottom Left, WT mice treated with vehicle. **e,** Proposed model: PLpro released from infected cells in the airway epithelium and in the sensory ganglia activate Trpa1-expressing neurons to drive sneeze, pain, and the release of CGRP into the airways. Error bars and shading represent the mean ± standard error of the mean (SEM), *n* = biological replicates (animals).

bioRxiv preprint doi: https://doi.org/10.1101/2024.01.10.575114; this version posted January 11, 2024. The copyright holder for this preprint (which was not certified by peer review) is the author/funder, who has granted bioRxiv a license to display the preprint in perpetuity. It is made available under aCC-BY 4.0 International license.

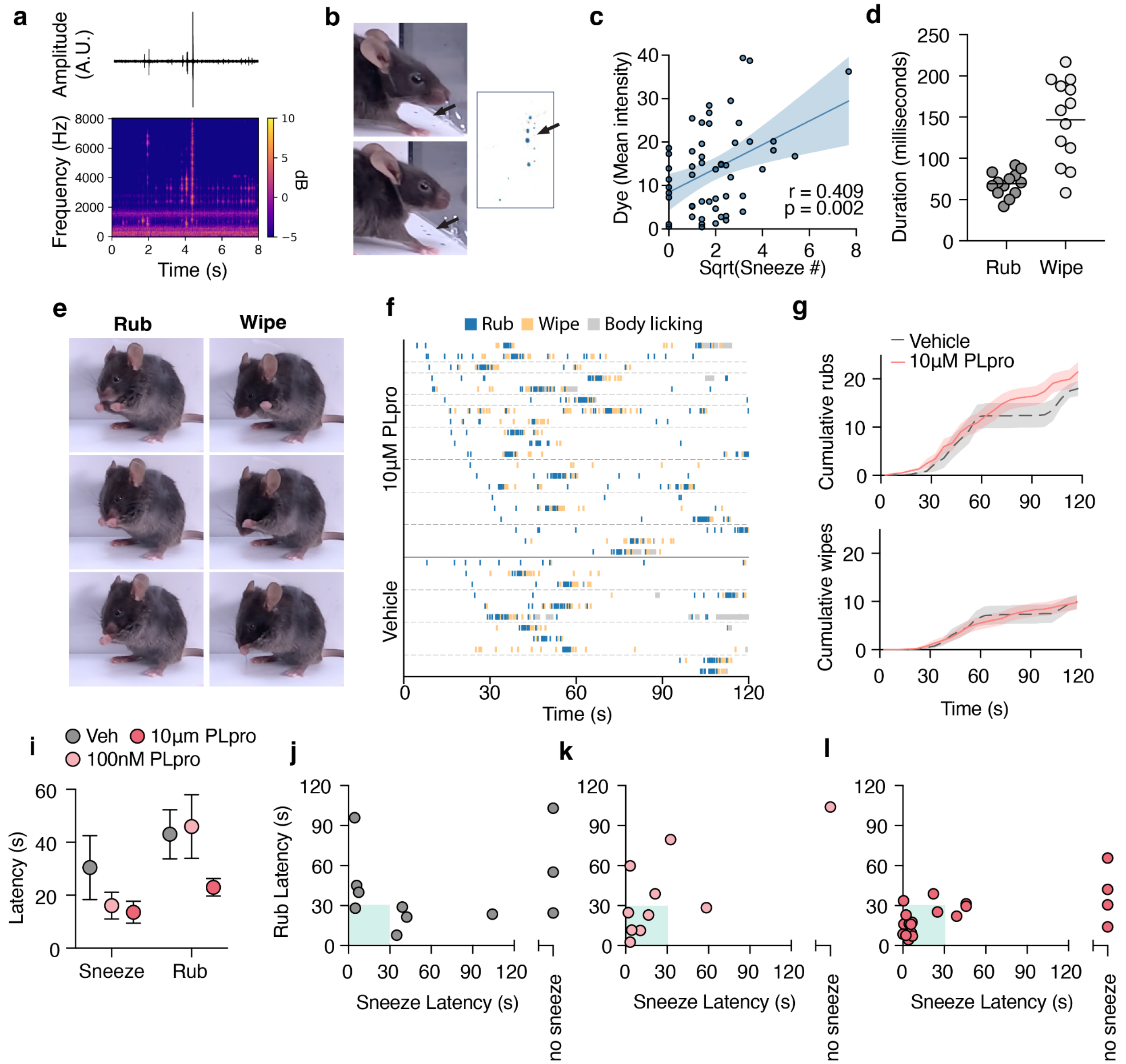

Supplemental Figure 1. Characterization of behaviors elicited by intranasal treatment.
a-c, Sneeze events are identified by audio waveforms and correlate to dye on the bottom of the chamber. **a,** Representative audio waveform during a sneeze **b,** Left, images during the same sneeze, arrows indicate dye from the sneeze on the bottom of the chamber. Right, image of dye expulsion from the sneeze event on the bottom of the chamber. **c,** The number of sneezes over 2 minutes are significantly correlated to the amount of dye at the bottom of the chamber. **d,** Duration of individual nose rub and face wipe events. **e,** Image sequence of a nose rub and face wipe. **f,** Raster plot of rubs, wipes, and body-licking events. **g,** Average cumulative rub and wipe counts from individual mice treated with 10 μM PLpro (n=20) or vehicle (n=11). **i-l,** Mean and standard error of the sneeze and rub latency and for individual mice treated with **j,** vehicle (n=11), **k**, PLpro (100 nM, n=12), and **l**, PLpro (10 μM, n=20), n = biological replicates (animals).

bioRxiv preprint doi: https://doi.org/10.1101/2024.01.10.575114; this version posted January 11, 2024. The copyright holder for this preprint (which was not certified by peer review) is the author/funder, who has granted bioRxiv a license to display the preprint in perpetuity. It is made available under aCC-BY 4.0 International license.

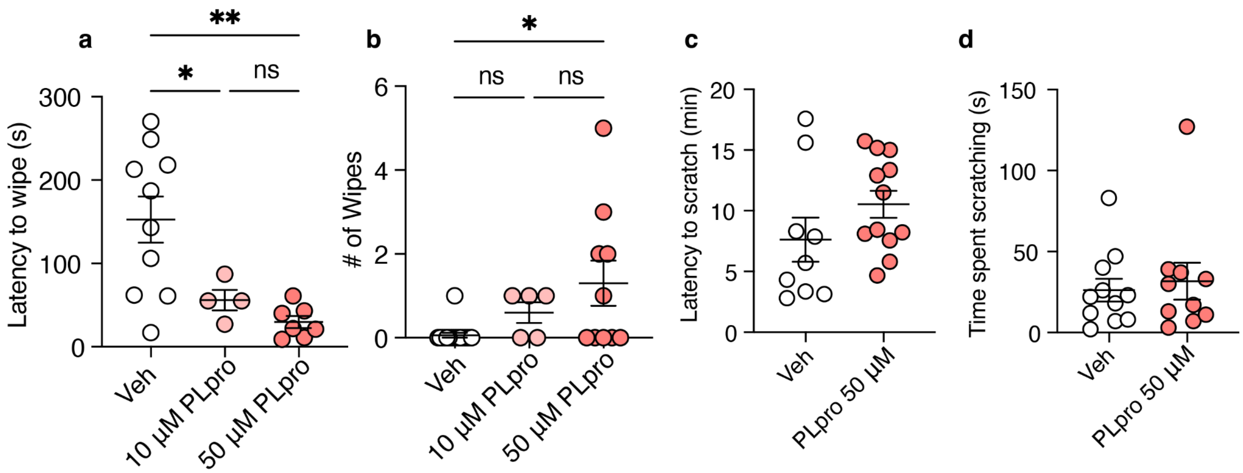

Supplemental Figure 2. PLpro triggers pain but not itch-associated behaviors.
a, PLpro elicited a shorter latency to the first wipe (One-way ANOVA: $p=0.003$, $F(2,18)=9.983$; Tukey's multiple comparisons, $p_{\text{Veh vs. 10µM PLpro}}=0.049$, $p_{\text{Veh vs. 50µM PLpro}}=0.003$, $p_{\text{10µM PLpro vs. 50µM PLpro}}=0.789$) and **b**, a dose-dependent increase in the number of wipes in the first minute. (Kruskal-Wallis: $p=0.0116$, $\chi2=8.920$, Dunn's multiple comparisons, $p_{\text{Veh vs. 10µM PLpro}}=0.134$, $p_{\text{Veh vs. 50µM PLpro}}=0.021$, $p_{\text{10µM PLpro vs. 50µM PLpro}}>0.999$), vehicle (*n*=17), PLpro (10 µM, *n*=5), PLpro (50 µM, *n*=10). **c-d,** PLpro does not elicit a shorter latency to scratching or any more scratching than vehicle injection, vehicle (*n*=11), PLpro (50 µM, *n*=10). Error bars represent the mean ± SEM. *n* = biological replicates (animals).

bioRxiv preprint doi: https://doi.org/10.1101/2024.01.10.575114; this version posted January 11, 2024. The copyright holder for this preprint (which was not certified by peer review) is the author/funder, who has granted bioRxiv a license to display the preprint in perpetuity. It is made available under aCC-BY 4.0 International license.

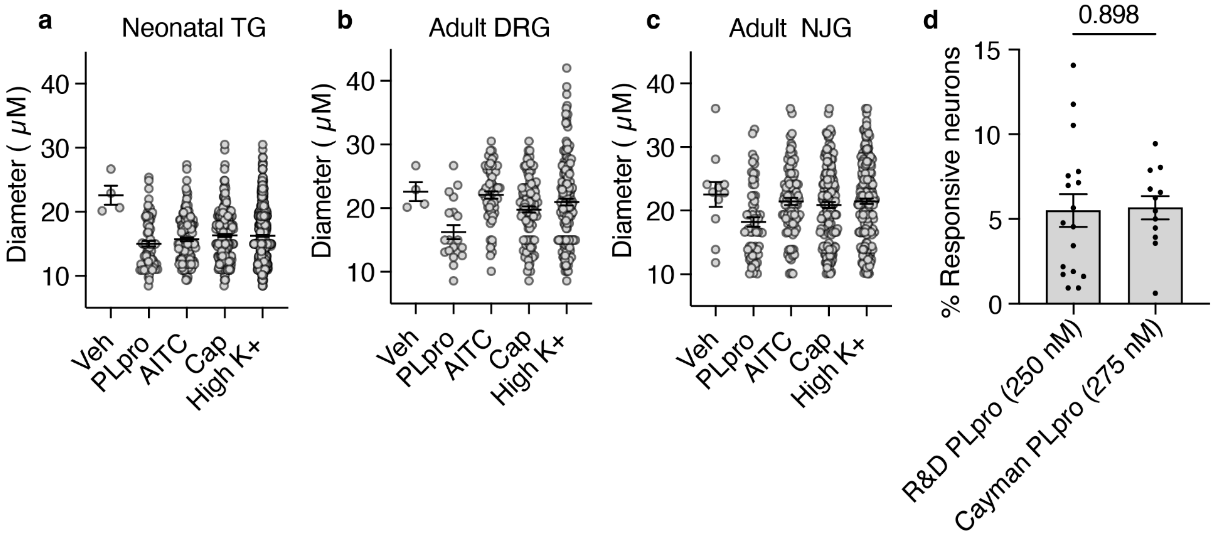

Supplemental Figure 3. PLpro activates small-diameter primary sensory neurons.
PLpro responders are of smaller diameter compared to vehicle responders and the population overall in **a.** neonatal mouse trigeminal ganglia (Veh: 22.6 μm, PLpro: 15.0 μm, AITC: 15.7 μm, Cap: 16.3 μm, High K+: 16.3 μm) **b,** adult DRG (Veh: 22.6 μm, PLpro: 16.2 μm, AITC: 22.1 μm, Cap: 19.8 μm, High K+: 21.0 μm) and **c,** adult NJG neurons (Veh: 22.5 μm, PLpro: 18.2 μm, AITC: 21.4 μm, Cap: 20.9 μm, High K+: 21.5 μm). **d,** PLpro from independent suppliers R&D Systems (#E-611, 5.5%, *n*=17) and Cayman (#31817, 5.7%, *n*=12) display no difference in the percent of responsive neurons in cultured TG, t-test: t=0.13, df=27, p=0.898. Error bars represent the mean ± SEM.

bioRxiv preprint doi: https://doi.org/10.1101/2024.01.10.575114; this version posted January 11, 2024. The copyright holder for this preprint (which was not certified by peer review) is the author/funder, who has granted bioRxiv a license to display the preprint in perpetuity. It is made available under aCC-BY 4.0 International license.

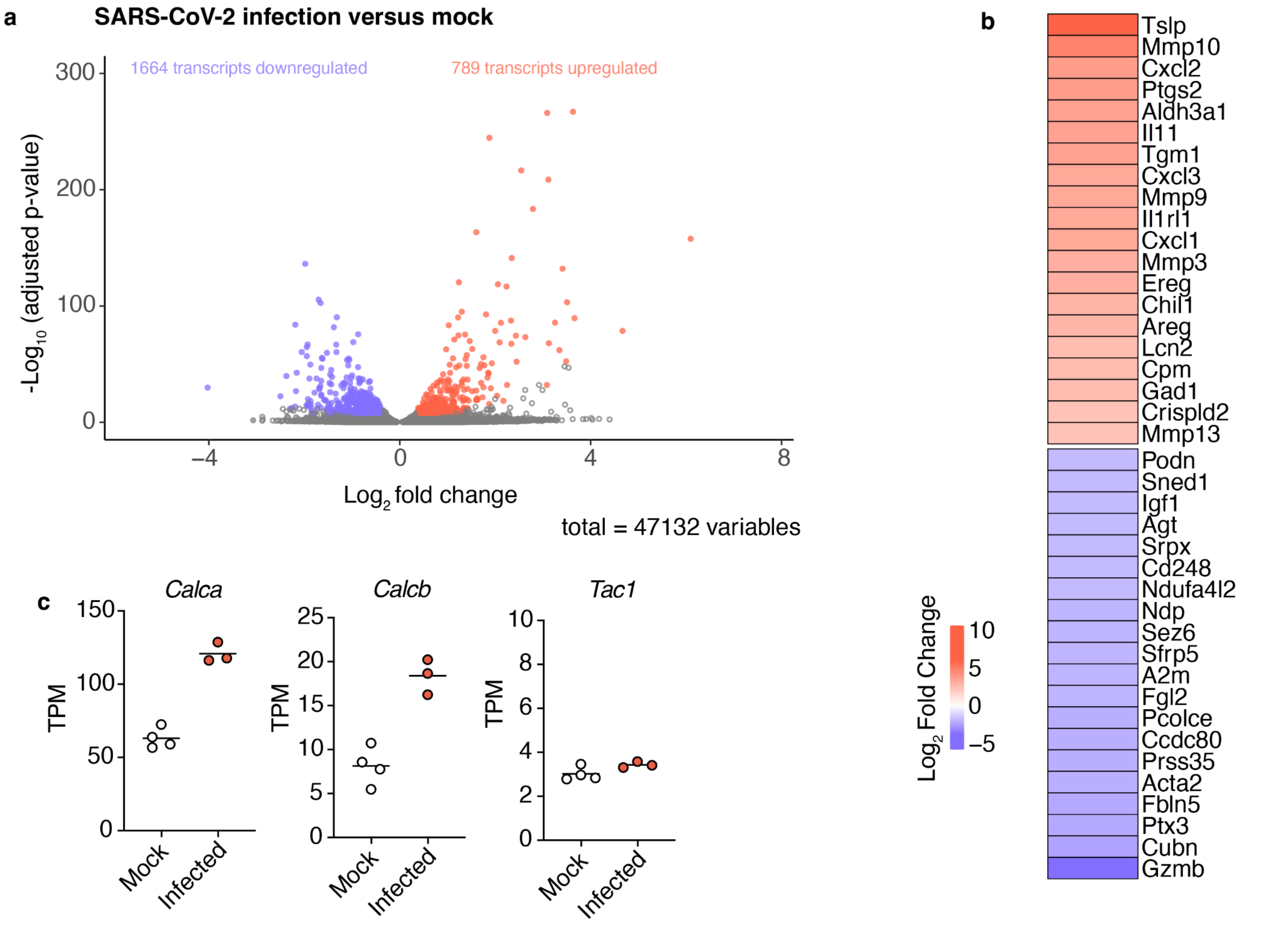

Supplemental Figure 4. SARS-CoV-2 infection drives gene expression changes in cultured primary mouse trigeminal ganglia.

a, 48 hours of SARS-CoV-2 infection upregulates 789 and downregulates 1664 transcripts compared to mock infection in cultured adult trigeminal ganglia, mock infected: *n*=4 wells, SARS-CoV-2 infected *n*=3 wells. Significant differentially expressed genes > 152 base mean, > 0.37 $\log_2$ fold change and an adjusted p-value < 0.001. **b,** Heatmap of the 20 most highly upregulated and downregulated transcripts ($\log_2$ fold change of infection vs mock). **c,** *Calca* and *Calcb* but not *Tac1* transcript counts (transcript per million [TPM]) are significantly upregulated by SARS-CoV-2 infection, *Calca*: adjusted $p_{\text{infection vs. mock}} = 9.05 \times 10^{-21}$, *Calcb*: adjusted $p_{\text{infection vs. mock}} = 5.52 \times 10^{-9}$, *Tac1*: adjusted $p_{\text{infection vs. mock}} = 0.991$.
